# Differential Cytokine and DNA Damage Response of Human Lung Tissue Models to Broad-Beam and Microbeam Radiotherapy

**DOI:** 10.3390/cells15060500

**Published:** 2026-03-11

**Authors:** Aleksandra Čolić, Marina Santiago Franco, Narayani Subramanian, Mabroor Ahmed, Susanne Raulefs, Jessica Müller, Stefan Bartzsch, Stephanie E. Combs, Thomas E. Schmid, Harry Scherthan

**Affiliations:** 1 Department of Radiation Oncology, TUM School of Medicine and Health, TUM University Hospital, Technical University of Munich, Ismaninger Straße 22, 81675 Munich, Germany; aleksandra.colic@tum.de (A.Č.); marina.franco@tum.de (M.S.F.); narayani.subramanian@tum.de (N.S.); mabroor.ahmed@tum.de (M.A.); susanne.raulefs@tum.de (S.R.); stefan.bartzsch@tum.de (S.B.); stephanie.combs@tum.de (S.E.C.); 2 Institute of Radiation Medicine (IRM), Helmholtz Zentrum München GmbH, German Research Center for Environmental Health, 85764 Neuherberg, Germany; 3 Bundeswehr Institute of Radiobiology affiliated to the University of Ulm, Neuherbergstr. 11, 80937 Munich, Germany; jessica4mueller@bundeswehr.org

**Keywords:** radiation, spatial fractionation, radiation pneumonitis, radiation fibrosis, 3D lung tissue model, TGF-β, collagen, pro-inflammatory cytokines

## Abstract

Radiotherapy (RT) is a standard treatment for lung cancer; however, radiation-induced toxicities such as pneumonitis and fibrosis limit dose escalation and tumor control. Therefore, improved RT approaches are needed. This study investigated the radiation response of human ex vivo normal lung tissue using the three-dimensional EpiAlveolar™ model. Tissue models were irradiated with broad-beam (BB) and two spatially fractionated microbeam radiation therapy (MRT) dose metrics: equivalent uniform dose (MRT-EUD) and valley dose (MRT-valley). Our findings show that ex vivo lung tissue is able to tolerate peak doses of 36 Gy following MRT-EUD. On day 21, models effectively repaired significant DNA double-strand break (DSB) damage seen in the MRT-EUD-irradiated peak regions. In contrast, persistent unresolved DSBs were detected in MRT-valley-irradiated models 21 days post irradiation. Prolonged culture time resulted in cell loss and a reduction in epithelial cell layers. A significant upregulation of the pro-inflammatory cytokine IL6 was observed in both BB and MRT-EUD groups at 21 days. Fibrotic collagen deposition was detected in one BB-irradiated model but was absent in remaining BB- and MRT-treated tissues. Further investigation is required to clarify the potential and suitability of EpiAlveolar™ models for studying radiation-induced lung injury.

## 1. Introduction

Lung cancer remains one of the leading causes of death worldwide, accounting for 2.48 million new cases and 1.82 million deaths in 2022 [1]. Approximately 80% of patients diagnosed with lung cancer have an evidence-based indication for radiotherapy (RT) at some point in their treatment [2]. Recent advances in technological capabilities such as four-dimensional computed tomography (4DCT), image guidance and stereotactic ablative radiotherapy (SABR) have significantly improved accuracy of treatments and survivability rates [3]. Despite the improvements and new technologies, one of the main limiting factors for an increased radiation dose and therefore an improved tumor control is the severe toxicity in the surrounding healthy tissue. With the lung being one of the most radiosensitive organs, the two major dose-limiting toxicities, radiation pneumonitis (RP) and radiation fibrosis (RF), significantly impact patients’ quality of life and contribute to morbidity, thus highlighting the need for further improvement in RT delivery [4]. Although pneumonitis is an acute condition and may be reversed, the continued attempt to repair the initial injury can ultimately lead to irreversible pulmonary fibrosis (PF) months after therapy [5]. The primary driver of fibrosis, transforming growth factor beta (TGF-β), causes the differentiation of fibroblasts into myofibroblasts that produce excess levels of collagen, thereby reducing lung elasticity and leading to loss of lung function [4,5]. A major source of TGF-β is M2 macrophages, whose infiltration and differentiation are stimulated following thoracic irradiation [6,7]. TGF-β initiates and maintains epithelial–mesenchymal transition (EMT), leading to activation of myofibroblasts and their expression of markers such as vimentin and alpha smooth muscle actin (αSMA) [8].

A promising step forward in treatment of lung cancers is a novel, still preclinical form of radiotherapy called microbeam radiation therapy (MRT), which has the potential to reduce damage to normal tissue. The concept of MRT, first proposed and defined in the 1990s by Slatkin and colleagues [9], is based on inhomogeneous dose distribution that is achieved with specifically designed collimators. Beam collimation allows for a typically homogeneous field to be spatially fractionated into quasi-parallel beams usually between 25 and 100 µm and with a center-to-center (CTC) distance of 200–400 µm. Such a dose distribution consists of “valley” (low-dose) regions, where the dose remains below the normal-tissue tolerance, and “peak” (high-dose) regions, where the latter dose often exceeds hundreds of Gray (Gy). Several groups have already shown that, compared to conventional broad-beam (BB) radiation, MRT is better tolerated by the healthy tissue, and it is more effective in improving survival, tumor growth delay and tumor control [10,11,12]. Most studies investigating radiation responses of the lung ex vivo are conducted on two-dimensional (2D) monolayer cultures of specific cell types, which lack a complex microenvironment and do not fully represent the organ/tissue physiology [13,14]. Alternatively, animal models, primarily mice and rats used in radiation research, are endowed with their own challenges. Furthermore, respiratory motion in animal models can complicate positioning and targeting of the lung tumor precisely, therefore confounding a correct radiation treatment. Moreover, there is an increasing demand for the replacement, reduction and refinement (the 3Rs) of animal experiments [15].

In recent years, three-dimensional (3D) tissue models have served as a bridge between in vitro 2D cell models and in vivo animal models. The 3D environment allows for particular cell types of a tissue to closely interact with each other in their three-dimensional environment, offering a closer representation of tissue architecture and biological responses [16]. In a study conducted on lung organoids, changes associated with radiation-induced fibrosis could be observed within 4–7 days [17]. In the present study, we utilized a commercially available, human 3D co-culture model of the lung air–blood barrier (EpiAlveolar™, MatTek Corporation, Ashland, MA, USA), consisting of primary human alveolar epithelial cells type 1 (AT1) and 2 (AT2), pulmonary endothelial cells and fibroblasts. The cells are grown at the air–liquid interface, which closely mimics the conditions in the human lung. In this study we investigated the spatial distribution of DNA double-strand break (DSB) damage induced by BB and MRT irradiation at subcellular resolution, as well as their impact on TGF-β and epithelial–mesenchymal transition (EMT) markers associated with the onset of fibrosis. Finally, we scrutinized the expression levels of cytokines linked to inflammation. This study provides novel insights into the distinct radiation responses elicited by BB and MRT irradiation in human 3D lung tissue models.

## 2. Materials and Methods

### 2.1. 3D Lung Tissue Model

EpiAlveolar™ human 3D lung tissue models were obtained from MatTek Life Sciences (MatTek Corporation, Ashland, MA, USA). The 3D structure is composed of primary human AT1 and AT2 cells, as well as primary fibroblasts, with both cell types being grown on the apical side of a modified semipermeable insert membrane, while the pulmonary endothelial cells are seeded on the basal side [18]. We furthermore enriched this 3D lung tissue model with the addition of THP-1 macrophages (ATCC TIB-202™, Manassas, VA, USA).

Upon arrival from the manufacturer, the tissues were individually placed in customized 6-well hanging culture plates with EpiAlveolar™ culture medium (MatTek Corporation, Ashland, MA, USA) and left to equilibrate overnight at 37 °C before irradiation on the following day. The medium was replaced regularly both on the apical surface and in the basal compartment following the manufacturer’s guidelines.

### 2.2. Irradiation Setup

Broad-beam irradiation was carried out on RS225 (X-Strahl Limited, Camberley, UK) with a 220 kVp X-ray spectrum filtered with 3 mm aluminum at a dose rate of 1.33 Gy/min. MRT irradiation was conducted using an XenX irradiation device (X-Strahl Limited, Camberley, UK). The MRT setup, previously developed by our research group [19], was equipped with a custom-made tungsten multislit collimator with a beam width of 50 µm and CTC distance of 400 µm. The peak dose rate was 4.5 Gy/min, while valley dose rate was 0.21 Gy/min, leading to a peak-to-valley dose ratio (PVDR) of 21. The beams were filtered with 3 mm aluminum. After irradiation, tissues were returned to the incubator (37 °C, 5% CO_2_) and cultured for either 3 or 21 days. A single dose of 3 Gy was delivered with BB irradiation, while for MRT irradiation two different approaches were used, the equivalent uniform dose (EUD) and the valley dose.

The EUD concept, proposed by Niemierko [20], is based on the linear quadratic (LQ) model and it assumes that different dose distributions are equivalent if they lead to the same clonogenic cell survival. LQ model parameters, α and β, represent the radiation response of cell lines, tumors or tissues. In contrast, the valley dose is a physical dose metric where the valley dose matches the BB dose. It is more predictive of normal-tissue sparing as compared to other physical dose metrics (e.g., peak or average doses). However, it was shown to be not as predictive as the EUD, especially at low dose levels [21]. In this study, for the MRT-EUD approach, we used an α/β ratio of A549 (human lung adenocarcinoma) of 23.27 Gy (α = 0.419 and β = 0.018), previously reported by our research group [21]. The rationale behind using α/β values of A549 was that in the patient treatment setting, the normal surrounding tissue would be receiving the dose prescribed to the lung tumor. Although the biological response of normal tissue is not expected to be equivalent to that of tumor cells, evaluating the dose received by normal tissue at equal tumor control provides insight into the therapeutic window. With the MRT-EUD approach, the dose in the valleys was 1.69 Gy, while in the peaks it was 36.4 Gy. For the MRT-valley approach, the valley dose was 3 Gy, matching the BB dose, meaning that the peak dose was 63 Gy, significantly higher than with EUD irradiation. For each irradiation modality and timepoint, three individual models were irradiated. As controls, three sham controls were included for each timepoint.

### 2.3. Sample Collection and Tissue Processing

Tissues were collected on day 3 (D3) and 21 (D21) post irradiation (pIR). The EpiAlveolar™ models were washed twice with PBS and subsequently fixed with 4% formaldehyde for 30 min. Fixed samples were then once again washed with PBS. A 20-gauge needle (B.Braun, Melsungen, Germany) was used to induce two symmetrical punches in the model membrane to indicate the orientation of MRT irradiation for proper embedding. Tissue membranes were cut from the insert using a scalpel (B.Braun, Melsungen, Germany) followed by embedding in 2% low-melting agarose and allowed to harden. Samples were then processed, and paraffin embedded in blocks using a Tissue-Tek VIP^®^ 6 tissue processor (Sakura Finetek Europe B.V., Alphen aan den Rijn, The Netherlands).

### 2.4. Immunofluorescence Staining (IF)

Paraffin tissue sectioning of the EpiAlveolar™ models and immunostaining was done as described by Scherthan et al. [22]. Briefly, 5 µm sections were cut from paraffin blocks perpendicularly to the MRT irradiation pattern, using a Leica RM2255 microtome (Leica Biosystems, Nußloch, Germany), mounted on super-frost plus slides (Carl Roth, Karlsruhe, Germany) and dried overnight at 60 °C. Sections were de-waxed in xylene and rehydrated in a graded series of alcohols. Antigen retrieval was performed by submersing the slides for 45 min in sodium citrate at 95 °C followed by gradual cooling to ~37 °C. Non-specific protein binding was blocked for 10 min at 37 °C using TCTG (TRIS, 5% Na-Casein, 0.1% Tween20, 0.1% fish-gelatin) buffer. The slides were incubated for 1.5 h at 37 °C with the primary antibodies against γH2AX, 53BP1, alpha smooth muscle actin (αSMA), vimentin and collagen type 1 alpha 1 (COL1A1) (Table 1) in TCTG buffer, subsequently washed in TCTG and incubated with the secondary antibodies (Table 1) for 1 h at 37 °C. Finally, slides were washed in TCTG, stained with DAPI (Carl Roth, Karlsruhe, Germany) in PBS for 2 min and mounted in ProLong Glass antifade (Invitrogen, Fisher Scientific, Schwerte, Germany).

### 2.5. Microscopy and Quantitative Image Analysis

Fluorescent images of stained 3D lung tissue were acquired with the automated digital imaging platform TissueFAXS (TissueGnostics, Vienna, Austria) as described previously [22]. Grayscale images of tissue cross-sections were acquired using a 40× lens on an Axio Observer Z1 fluorescence microscope (Zeiss, Oberkochen, Germany). The TissueQuest analysis software package (TissueGnostics, Vienna, Austria; version 7.1.1.141) was used to generate contour masks for nuclei (DAPI-positive cells) and to quantify DSB foci (γ-H2AX and 53BP1 foci within positive cells) as well as collagen-, vimentin- and αSMA-positive cells (fibrosis-associated). DSB foci were enumerated within the nuclear contour masks with the StrataQuest software package (TissueGnostics, Vienna, Austria; version 7.1.1.141). Previously published work describes the principle and methods behind the applied algorithms [23]. Individual nuclei were assigned by a position annotation algorithm along each lung tissue cross-section, which allowed visualization of the spatial distribution of subcellular DNA damage and lung cell types across the models. In some instances, when parts of the lung tissue were curved or gaps were present, software-assisted linear alignment (TissueGnostics, Vienna, Austria; version 7.1.1.141) was employed to digitally straighten these sections. For this, a start point was interactively marked at one end of the section and set as the relative starting point for the relative annotation of the nuclei positions, which were then projected linearly in 2D. In cases in which a cross section was curved and/or displayed gaps on the slides, digital linear alignment led to a moderate increase in the cross-section length, while maintaining the relative spatial annotation of cells.

### 2.6. TGF-β ELISA

Supernatants from 3D EpiAlveolar™ tissue were collected at consecutive timepoints (D0, D1, D3, D6 and D21) and used to quantify the amount of secreted TGF-β. To this end, the Human TGF-β 1 DuoSet ELISA kit (R&D Systems, Minneapolis, MN, USA) was used according to the manufacturer’s protocol. Microplates were read at 450 nm and 540 nm (correction) wavelength with Infinite^®^ 200 PRO (Tecan, Männedorf, Switzerland).

### 2.7. Multiplex Inflammation Cytokine Assay

Medium from 3D lung tissues was collected at different timepoints (D0, D1, D3, D6 and D21) and used to determine cytokine concentrations. The concentration of inflammatory cytokines and chemokines—tumor necrosis factor alpha (TNFα), monocyte chemoattractant protein 1 (MCP1), interleukin 6 (IL6), and interleukin 8 (IL8)—was measured with LEGENDplex™ Human Inflammation Panel 1 (BioLegend, San Diego, CA, USA) according to the manufacturer’s instructions. Samples were individually acquired with a CytoFLEX (Beckman Coulter Life Sciences, Krefeld, Germany) flow cytometer and data were analyzed with the LEGENDplex Qognit software (legendplex.qognit.com; version 2025-05-01. © 2019 Qognit).

### 2.8. Statistical Analysis

All statistical analyses were conducted with GraphPad Prism version 9.5.0 (GraphPad Software, Boston, MA, USA). One-way analysis of variance (ANOVA) followed by Tukey’s multiple-comparison test was performed to test the differences between the groups, and a *p*-value of ≤0.05 was considered statistically significant. In cases where datasets did not pass one-way ANOVA assumptions, values were log-transformed before running the ANOVA analysis followed by Tukey’s test.

## 3. Results

### 3.1. MRT Irradiation Results in a Distinct Spatial Distribution of dsDNA Damage Across 3D Lung Tissue

**Figure 1:**
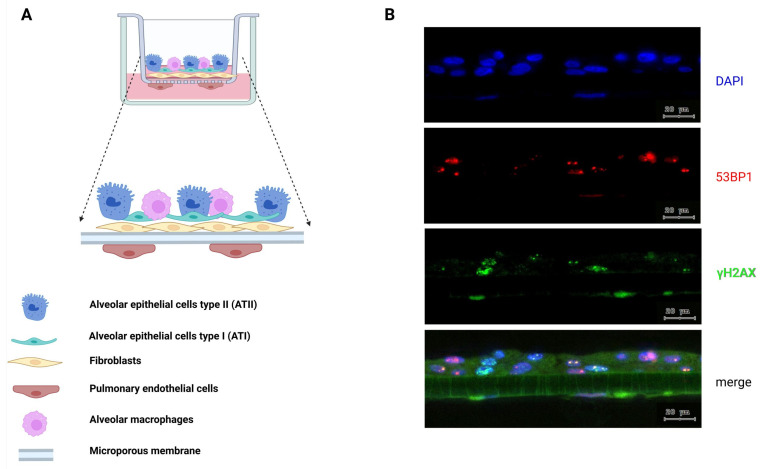
Schematic figure and immunofluorescence DNA damage images of a D3 lung model. (**A**) Illustration showing different cell types and their position in the tissue culture insert. (**B**) Representative image of 53BP1 and γH2AX DSB damage foci on an MRT-valley-irradiated 3D lung tissue on D3 pIR; blue: DAPI – nuclei; red: Cy3 – 53BP1; green: Cy5 – γH2AX. The merged image was increased in brightness for the green γH2AX channel to reveal the autofluorescence of the lung tissue model for visualization of the tissue support membrane below the multicellular epithelium. Scale bar = 20 µm. (**A**) Created with BioRender.

Lung model tissue cross-sections were stained for the DNA DSB-damage-specific markers 53BP1 and γH2AX (Figure 1B) and analyzed as previously described [22].

**Figure 2:**
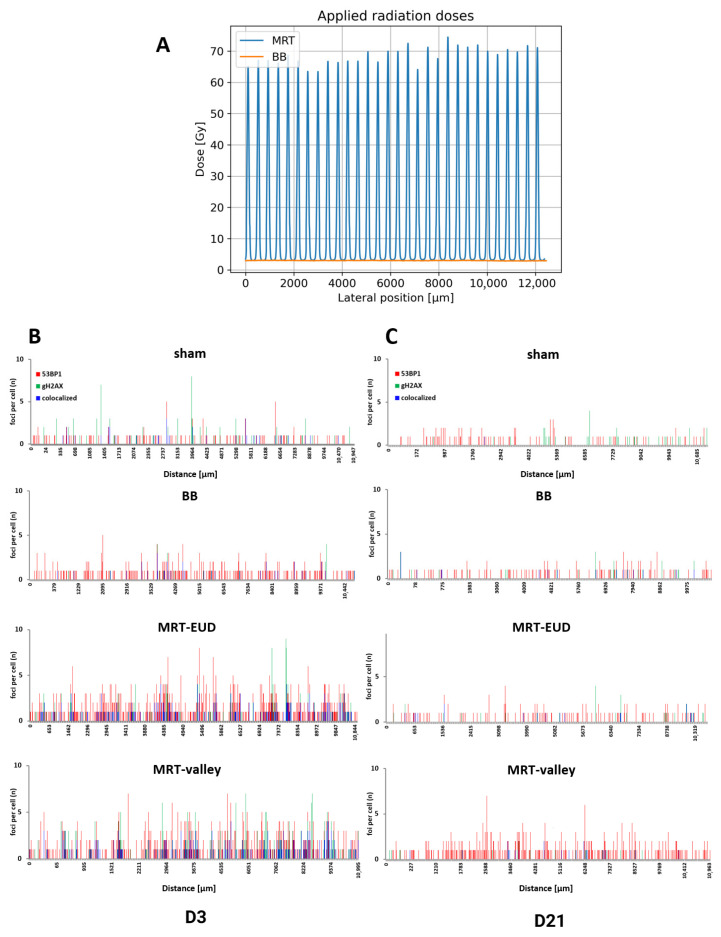
BB and MRT irradiation dose profiles, with representative graphs of DSB foci distribution per individual cell nucleus (height of lines represents foci no. of each individual cell) according to their position along the 3D lung tissue cross-sections. (**A**) Dose profiles for MRT (blue) and BB (orange) irradiation correlate with distribution of DSB damage load. (**B**) On D3 pIR, occasional 53BP1/γH2AX foci-positive cells were observed in sham-treated samples, while BB-irradiated cells exhibited more 53BP1 foci-positive cells with a relatively uniform distribution across the lung tissue sections. D3 MRT-exposed models (both EUD and valley) displayed distinct spatial accumulations of cells with a higher number of foci and colocalized foci indicating the positions of irradiation peaks. (**C**). On D21 pIR, most of the DNA damage was repaired in BB- and MRT-EUD-irradiated samples, while MRT-valley tissues still displayed a high frequency of 53BP1-positive cells; for FPC values, see Section 3.2. Enlarged details for D3 and D21 spatial foci distribution graphs are shown in Supplementary Figures S1 and S2.

Sham-irradiated tissues displayed a low number of randomly distributed cells positive for 53BP1 and/or γH2AX foci on D3 pIR. After 3 Gy BB irradiation, cells with 53BP1 foci were frequent and uniformly distributed across the lung models (Figure 2B).

Both MRT-EUD and MRT-valley irradiation led to defined patterns of DSB foci distribution (Figure 2B). The distribution of cell clusters positive for both 53BP1 and γH2AX foci (colocalized cells; Figure 2B) could be correlated with CTC distances of MRT peaks (Figure 2A). The minimal difference in distances can be attributed to variations such as slight misorientation of the tissue during embedding/cutting and digital tissue straightening. At D21 pIR, DSB damage was repaired in BB- and MRT-EUD-irradiated samples, with 53BP1 and γH2AX foci distribution resembling that of sham-irradiated samples (Figure 2C). In contrast, even on D21 pIR, MRT-valley-irradiated tissues exhibited a higher number of cells positive for 53BP1 foci (Figure 2C). The average number of 53BP1 foci per cell (FPC) on D21 was 0.18 (±0.02 SEM) for sham, 0.14 (±0.02) for BB, 0.22 (±0.03) for MRT-EUD and 0.51 (±0.03) for MRT-valley.

### 3.2. D21 MRT-Valley-Irradiated Models Display Persistent 53BP1 Foci

**Figure 3:**
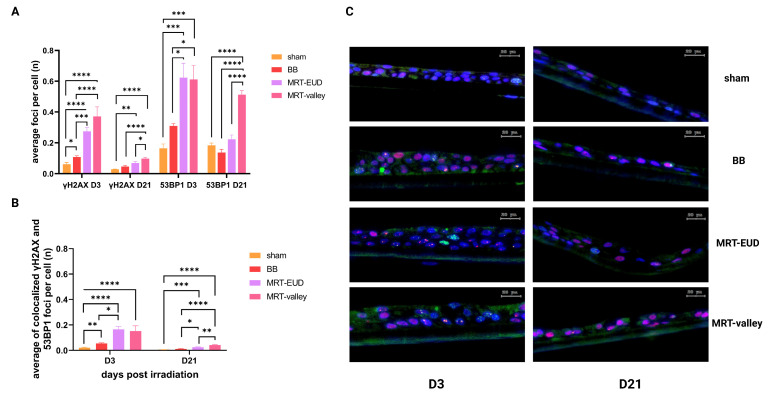
Quantification of γH2AX and 53BP1 foci on D3 and D21 pIR. (**A**) MRT-EUD and MRT-valley induced a higher number of DSB FPC at D3 pIR than BB irradiation. At D21 pIR the average number of 53BP1 FPC in BB and MRT-EUD tissues was similar to sham-irradiated tissues, while with MRT-valley a high number of cells with foci was still present. (**B**) The average number of colocalized foci was highest in MRT-irradiated samples on both D3 and D21 pIR. (**C**) Representative IF images of γH2AX and 53BP1 foci in each irradiation group on D3 and D21 pIR. Blue: DAPI-nuclei; red: Cy3-53BP1, green: Cy5-γH2AX. Cells with entirety of nuclei labeled in green represent pan-γH2AX cells. Scale bars = 20 µm. Data are presented as the mean ± SEM of nine technical replicates, coming from three biological replicates (except for MRT-EUD γH2AX D21 were *n* = 8). * *p* < 0.05; ** *p* < 0.01; *** *p* < 0.001; **** *p* < 0.0001.

On D3 pIR, all the irradiation modalities led to an increase in the average number of γH2AX foci per cell compared to the non-irradiated tissue. BB-irradiated models (3 Gy) displayed an average of 0.11 FPC (*p* < 0.05), while MRT-EUD (1.69 Gy valley/36.4 Gy peak) and MRT-valley (3 Gy valley/63 Gy peak) irradiation induced a significantly higher number of FPC, on average 0.27 (*p* < 0.0001) and 0.37 (*p* < 0.0001) respectively, as compared to the sham control (0.06 FPC). A similar trend could be observed for the average number of 53BP1 FPC on D3 pIR; however, the average number of 53BP1 FPC following MRT-EUD and MRT-valley irradiation was higher than those of γH2AX (0.62 (*p* < 0.001) and 0.61 (*p* < 0.001), respectively; Figure 3A). In comparison to BB irradiation (IR) on D3 MRT-EUD and MRT-valley had significantly higher average γH2AX FPC (*p* < 0.001 and *p* < 0.0001 respectively). Both MRT approaches also induced significantly (*p* < 0.05) higher average numbers of 53BP1 FPC on D3 compared to those of BB irradiation (0.31 FPC) (Figure 3A). On D3 the percentage of cells displaying DNA damage (one or more 53BP1/γH2AX foci per cell) was highest in MRT-irradiated samples (Table 2).

On D21 pIR, most of the γH2AX damage was repaired. For BB, the average number of γH2AX FPC (0.05) returned to a basal level, similar to that of sham samples, while a significant amount of foci persisted in lung models irradiated with MRT-EUD (0.07, *p* < 0.01) and MRT-valley (0.1, *p* < 0.0001) (Figure 3A), as compared to the control (0.03). At D21, MRT-valley maintained a higher number of average γH2AX FPC compared to both BB and MRT-EUD (*p* < 0.0001 and *p* < 0.05 respectively) (Figure 3A). Accordingly, MRT-valley displayed the highest percentage of cells with focal damage (35% of cells had 53BP1 foci and 8% γH2AX foci) (Table 2).

The average number of 53BP1 FPC on D21 pIR returned close to basal levels for both BB (0.14, *p* > 0.05) and MRT-EUD (0.22, *p* > 0.05). MRT-valley-irradiated tissues retained a significantly higher average number of 53BP1 FCP (0.51, *p* < 0.0001) than those of sham samples (0.18), as well as compared to BB and MRT-EUD (*p* < 0.0001).

In some cases, cells were positive for both γH2AX and 53BP1 foci, and such foci were addressed as colocalized (Figure 3B). At D3 pIR, BB-treated samples had an average of 0.05 colocalized FPC (*p* < 0.01) while MRT-EUD and MRT-valley had 0.17 (*p* < 0.0001) and 0.15 (*p* < 0.0001) respectively, as compared to the control (0.02). When compared to BB on D3, MRT-EUD induced a larger number of DSBs, represented by colocalized foci (*p* < 0.05). On D21 pIR the average number of colocalized FPC was minor, but still significantly higher in MRT-irradiated samples (0.03, *p* < 0.001 for MRT-EUD and 0.04, *p* < 0.0001 for MRT-valley), as compared to the control (0.005), as well as compared to BB (0.01) (*p* < 0.05, *p* < 0.0001 respectively) (Figure 3B).

### 3.3. Irradiation and Culturing of Lung Models Induces Cell Loss

**Figure 4:**
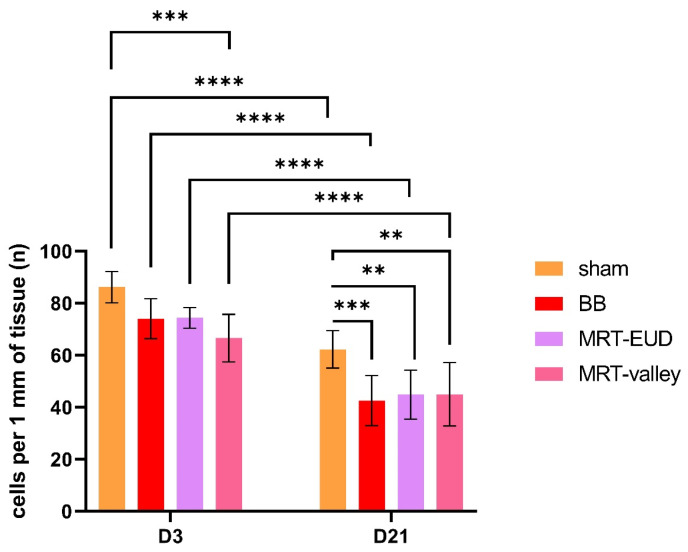
Cell numbers/mm model cross-section on D3 and D21. On D3, only MRT-valley had a significantly lower number of cells/mm compared to the sham. On D21, all radiation treatments displayed a significantly reduced number of cells/mm as compared to the sham tissues. Both radiation and tissue culture for 21 days led to a significant cell loss on D21 when compared to D3. Data are presented as the mean ± SD of nine technical replicates, coming from three biological replicates; ** *p* < 0.01; *** *p* < 0.001; **** *p* < 0.0001.

On D3, MRT-valley irradiation led to a significant (*p* < 0.001) reduction in cell numbers compared to the sham. While the sham had an average of 83.17 cells per mm of tissue, for MRT-valley it was 66.6 cells/mm. On D3, BB and MRT-EUD had on average 74.07 and 74.36 cells/mm respectively, with no significant reduction in cell numbers as compared to the sham (*p* > 0.05) (Figure 4). On D21, all radiation groups had significantly lower number of cells compared to the non-irradiated sham. BB-irradiated samples (42.58 cells/mm, *p* < 0.001) displayed lowest number of cells compared to the sham (62.24), while the tissues irradiated with MRT-EUD and MRT-valley displayed similar number of cells/mm (44.87 and 44.94 respectively; both *p* < 0.01 relative to sham). There were no significant differences in cell numbers between MRT- and BB-irradiated samples on D21. When compared to D3 sham, all D21 irradiated tissues, as well as D21 sham, had significantly lower numbers of cells/mm (*p* < 0.0001). Additionally, from D3 to D21, a significant reduction in cell numbers can be observed within each radiation group (*p* < 0.0001) (Figure 4). The average 27.77% loss of total cell number/mm observed in D21 sham can be attributed to the prolonged cell culture time, while the irradiation-induced cell loss amounted to an additional 22.82% for BB, 20.16% for MRT-EUD and 20.08% for MRT-valley (Table 3), the differences being insignificant among the irradiation modalities (Figure 4).

### 3.4. Similar TGF-β Levels After BB and MRT Irradiation

TGF-β is a known profibrotic cytokine involved in the onset of pulmonary fibrosis through the process of EMT [24]. Slight fluctuations in TGF-β concentration of radiation-treated samples were observed throughout different timepoints. Despite slight changes from one timepoint to another, no significant difference in the amount of secreted TGF-β was observed when comparing BB- and MRT-irradiated tissues to the sham-treated samples (Supplementary Figure S3).

### 3.5. Similar Expression of Fibrosis-Associated Markers After BB and MRT Irradiation

**Figure 5:**
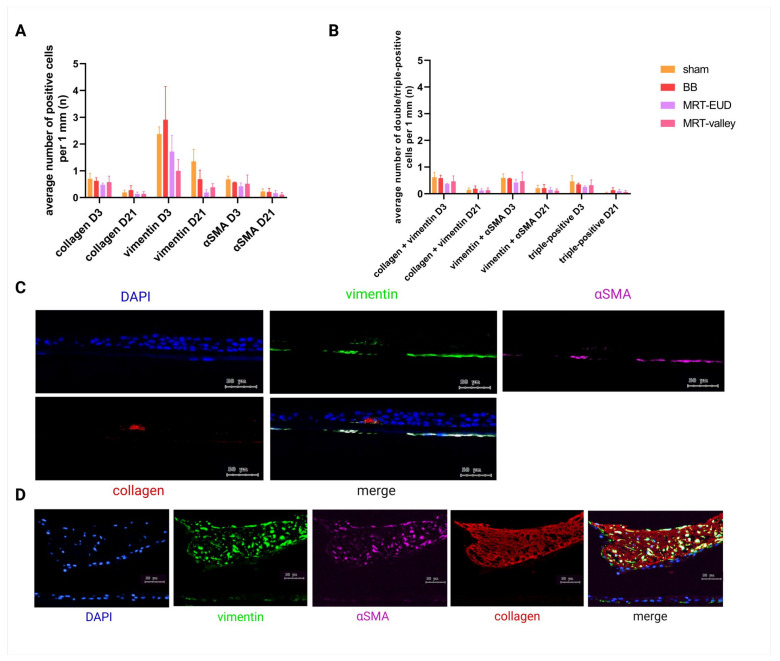
Quantification of EMT-marker-positive cells. Collagen-, vimentin- and αSMA-positive cells. Vimentin-positive cells were the most prevalent type of cells for all groups on D3 pIR (**A**). Although the average number of vimentin-positive cells per mm cross-section was reduced by MRT-EUD and MRT-valley IR, these differences are non-significant. The number of double- or triple-positive cells remained low and insignificant at both timepoints for BB and MRT treatments (**B**). Representative IF images of EMT markers (**C**). BB-irradiated model displaying extensive collagen deposition and vimentin + αSMA-double-positive cells (**D**). Blue: DAPI-nuclei, green: AF488-vimentin, violet: Cy5-αSMA; red: Cy3-collagen. Scale bars = 50 µm. Data are presented as the mean ± SEM of three biological replicates. Enlarged details of (**D**) are presented in Supplementary Figure S4.

Pulmonary fibrosis is characterized by EMT, a process regulated by TGF-β, in which epithelial cells, most notably AT2 cells, change their morphology and acquire mesenchymal markers such as vimentin and α smooth muscle actin. Such changes are followed by an increased deposition of collagen [25]. To investigate the effect and extent of radiation impact on EMT, we performed IF staining for the markers mentioned above (vimentin, αSMA and collagen). First, we looked at cells that were positive only for a single marker, and although vimentin-positive cells were the most abundant for both sham-treated and irradiated tissues, there was no significant difference in their numbers between the treatment groups (*p* > 0.05). The same trend was observed for other markers both on D3 and D21 pIR (Figure 5A). While the number of positive cells was noticeably lower by D21 pIR (Figure 5A), this was not statistically significant (*p* > 0.05) relative to D3. As shown in Section 3.3, a loss in cell number occurs between D3 and D21 in sham-treated tissues and in all irradiation groups. Additionally, we looked for cells which were either positive for two markers or positive for all three markers. Altogether, only a few such cells were counted across the models, with no significant difference between the groups (*p* > 0.05; Figure 5B). Representative images of EMT markers and collagen are presented in Figure 5C. Interestingly, we observed one BB-irradiated tissue model with a nodule of extensive collagen deposition on D3 pIR, which contained an increased number of cells expressing both αSMA and vimentin (Figure 5D) reflecting a fibrosis-type reaction.

### 3.6. IL6 Secretion Is Upregulated on D21 After BB and MRT-EUD Irradiation

**Figure 6:**
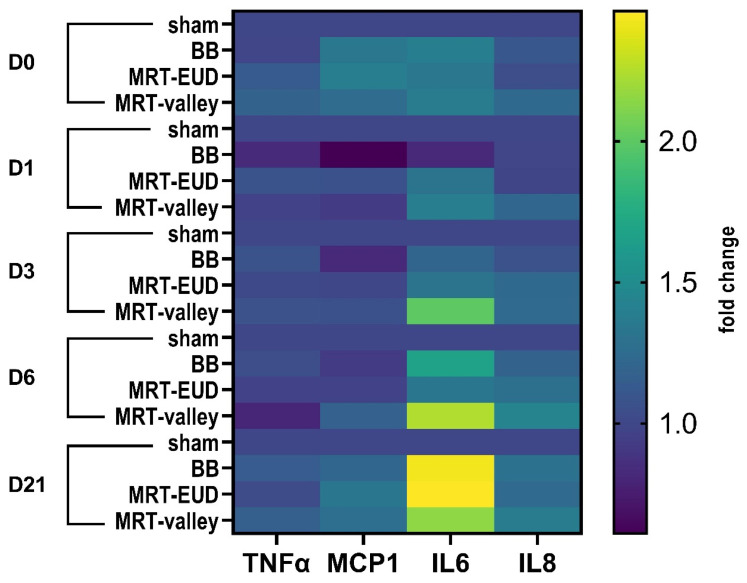
Heatmap illustrating the regulation of inflammation-associated cytokines on irradiated 3D lung tissue. Only IL6 was significantly upregulated on D21 following BB and MRT-EUD irradiation. MRT-valley showed an upregulation trend for IL6 on D3, D6 and D21. Data are presented as an average fold change (fc) of three biological replicates (except for MRT-EUD D1/D21 IL8 and MRT-EUD D21 MCP1, where *n* = 2).

Radiation-induced pneumonitis is an acute event characterized by injury to the lung epithelium and vascular endothelial cells. Such injury leads to alveolar epithelial cell damage and subsequent cytokine and chemokine release from infiltrated immune cells [5]. On D1, D3 and D6, no statistically significant differences in cytokine concentrations were observed for any of the treatment groups. On D21, neither BB nor MRT irradiation had a significant impact on TNFα secretion, and the same trend was observed for IL8 and MCP1 (*p* > 0.05). However, BB and MRT-EUD had a significant (*p* < 0.05) effect on IL6 secretion on D21 pIR, with average fold changes (fc) of 2.43 and 2.46, respectively. A trend could be observed with MRT-valley, where IL6 was upregulated on D3, D6 and D21 (fc of 2, 2.24, 2.15 respectively); however, this was not statistically significant (*p* = 0.44, *p* = 0.14 and *p* = 0.22 for the respective timepoints) (Figure 6).

## 4. Discussion

RT plays a crucial role in cancer treatment for patients with inoperable lung tumors. However, complications in the irradiated surrounding healthy tissue, such as radiation pneumonitis and radiation fibrosis, still pose a major issue for potential dose escalation and subsequent improvement of tumor control [4,5]. Despite recent technological advancements, there is still a need to further improve current radiotherapy approaches [26].

Ionizing radiation is known to induce DSBs, a lethal type of DNA damage that results in genomic instability, which, if not repaired, can lead to cell death or carcinogenesis [27]. DSBs initiate a DNA damage response (DDR) in which a number of responsive proteins are recruited for pathway choice and DSB repair. Among these proteins are γH2AX and 53BP1, which can be visualized by immunofluorescence as radiation-induced foci (RIF) [28,29]. We investigated how BB irradiation and two different MRT approaches (MRT-EUD and MRT-valley) interact with healthy lung tissue in a human ex vivo model. Sham-treated tissues displayed low numbers of γH2AX and 53BP1 DSB foci-positive cells, both on D3 and D21 after the treatment. On D3 we observed a uniform distribution of RIF across the tissue models treated with BB. Meanwhile, both MRT-EUD and MRT-valley models had distinct accumulations of cells with high DSB foci numbers, with the distance corresponding to the peak dose regions of our MRT radiation geometry. Overall, there were more cells with 53BP1 DSB foci than γH2AX foci per nucleus. This resulted in a reduced percentage of the 53BP1 foci colocalizing with γH2AX foci. Colocalizing foci were frequently observed in the high-dose peak regions on D3 pIR. Similar observations were made in proton MRT-irradiated epidermis models [22], in which most damage-carrying cells displayed 53BP1 foci only. Persistent 53BP1 DSB foci at later timepoints post irradiation have also been reported in other cell types [30]. As differentiated tissues primarily consist of non-cycling G1-phase cells, they likely rely on 53BP1 as a DNA damage reader and signaling factor that promotes NHEJ in this context [31]. Both γH2AX and 53BP1 foci are formed within minutes following DNA breakage, usually between 15 and 30 min, and persist for several hours [32]. Scherthan and colleagues (2022) observed that human epidermis models treated with proton minibeam radiotherapy (pMBRT) exhibited sharp localization of massive pan-γ-H2AX DNA damage in 66 µm pMBRT peak regions 0.5 h and 6 h after 27 Gy proton irradiation, while at D3 the damage pattern had receded to focal regions in the affected nuclei that were scattered across the models [22]. In contrast, while we clearly observed DNA damage on D3 pIR, we were still able to detect distinct clusters of cells with elevated focal damage, representing the parts of the tissue that were exposed to high peak-region doses of MRT irradiation. These findings suggest that dose–volume effects may induce delayed DSB repair in 3D lung tissue and/or that the high doses applied to the peaks (63 Gy) lead to chromatin regions harboring complex DSBs that are refractory to repair [33].

At D21, the number of RIF per cell in BB and MRT-EUD samples resembled that of sham-treated models, indicating that most of the DNA damage was successfully repaired. In contrast, tissues irradiated with MRT-valley retained a higher number of cells positive for 53BP1 foci. Furthermore, the average count of 53BP1 foci per cell was also higher than for γH2AX foci. It has been widely reported that a subclass of RIF can persist for several days or weeks, and as such are a consequence of complex DNA damage, which forestalls the completion of repair [34,35,36]. Ahmed and coworkers (2012) reported that high doses of 50 Gy γ-irradiation on a minipig skin led to the formation of RIF persisting up to 70 days post treatment, due to impaired DSB repair [37]. These findings are in line with our results, considering that with MRT-valley irradiation the dose in the peak regions was 63 Gy, considerably higher than the MRT-EUD peak dose of 36 Gy, and higher than the 27 Gy peak dose in the pMBRT irradiation of ex vivo epidermis models [22].

Continuous DDR signaling can be a trigger for cellular senescence, a state in which cells enter a permanent cell cycle arrest and this interconnection has been previously reported by Isermann and coworkers (2020) [38]. Moreover, senescence is accompanied by secretion of specific pro-inflammatory cytokines and chemokines, termed senescence-associated secretory phenotype (SASP) [39]. Senescence in AT2 cells depletes the stem cell pool, impairs the tissue repair and ultimately plays a critical role in onset of pulmonary fibrosis [40]. Taken together, our results suggest that a dose of 36 Gy in the peaks can be well tolerated by the lung tissue. Moreover, we show that DNA damage at D21 in BB and MRT-EUD irradiated 3D lung tissue models was repaired to DSB foci numbers and distribution patterns resembling that of sham-irradiated control models.

Following these results, we further investigated how radiation affects the cell numbers. On D3, MRT-valley-irradiated tissues induced the highest cell loss relative to sham irradiation, which could be explained by the highest dose in its peak regions (63 Gy). Comparing cell numbers between D3 and D21 revealed a significant cell loss of ~28% between sham tissues at these timepoints. Radiation exposure induced an additional ~20% cell loss in the irradiated models at D21, with the difference in cell numbers between the irradiation groups being insignificant. The culture- and irradiation-induced cell loss was accompanied by a clear reduction in epithelial height between D3 and D21 for sham-treated models and for the irradiated ones. These findings indicate that future research on the proliferation response, irradiation-induced senescence and cell death in response to MRT irradiation in 3D EpiAlveolar™ models should implement more timepoints.

One of the key processes of radiation-induced pulmonary fibrosis is the differentiation of fibroblasts with epithelial phenotype into myofibroblasts, which are characterized by mesenchymal phenotype. This event, named EMT, is driven mainly by TGF-β [25]. As such, we sought to understand whether the investigated radiation treatments induced a change in TGF-β secretion. Neither of the radiation modalities were able to trigger significant changes in TGF-β concentrations, independent of the timepoints studied post irradiation. The 3 Gy applied with BB and 3 Gy in the valleys of MRT-valley treatment, as well as 1.69 Gy in the valley regions of MRT-EUD, may be below the threshold to upregulate TGF-β levels. In agreement, Yang and coworkers (2024) reported that a 3 × 6 Gy fractionated radiation regime was also insufficient to trigger a change in TGF-β secretion in cancer-associated fibroblasts (CAFs) [41]. Conversely, the higher doses in the peak regions of MRT-EUD and MRT-valley (36 Gy and 63 Gy respectively) would be expected to elicit a fibrotic response, as 20 Gy has been reported to induce lung fibrosis in mouse models [42,43]. However, exploiting the dose–volume effect, our results suggest that 3D lung tissues are able to effectively tolerate the high peak doses applied.

AT2 cells have also been shown to acquire mesenchymal phenotype, contributing to the pool of myofibroblasts and extracellular matrix (ECM) deposition [44]. Therefore, we investigated how mesenchymal markers such as vimentin and αSMA are regulated following radiation. Additionally, we looked at collagen, which is secreted by myofibroblasts, and as such ultimately damages lung structures, contributing to fibrosis. We observed no significant difference in number of cells positive for αSMA, collagen and vimentin in any of the treated models, both on D3 and D21. Despite higher doses in the peaks, MRT-EUD and MRT-valley irradiation induced no significant difference in mesenchymal markers, nor did they lead to higher depositions of collagen. A similar observation was made for BB-treated models, except for one tissue model, in which we observed a fibrotic nodule. The nodule was characterized by a large deposition of collagen fibers containing vimentin- and αSMA-positive cells. Although there was fibrotic tissue formation in only one lung model, this indicates that human 3D EpiAlveolar™ models could be suitable for studying the fibrotic response. In agreement, Barosova and coworkers (2020) showed that tissues treated with TGF-β formed large nodules and had higher concentration of secreted COL1 [18]. In contrast, Trapetti and coworkers (2011) failed to observe fibrotic changes 6 months post MRT irradiation in mouse lungs [45]. Since fibrosis is a late effect of RT, it remains to be determined whether these findings extend to our human lung models, which were only cultured for 21 days.

Radiation-induced pneumonitis is an acute response that occurs in the first few days following radiation exposure [4]. RP is characterized by infiltration of immune cells such as macrophages and lymphocytes, followed by their release of pro-inflammatory cytokines including IL6, TNFα and IL8, as well as chemokines such as MCP1 [4,5,46]. TNFα, secreted primarily by activated macrophages, is an initiator of pro-inflammatory cascade [47]. It plays a role in development of fibrosis and induction of TGF-β [48]. Inhibiting TNFα has the potential to increase the likelihood of lung function preservation and hence leads to an overall better prognosis [49]. The human 3D lung models in our study secreted TNFα in very low concentrations independent of timepoint and radiation treatment. Irradiation with BB and MRT-EUD or MRT-valley did not result in a significant increase in TNFα. TNFα is also known to induce other cytokines, one of them being IL8. Produced by macrophages and epithelial cells, IL8 has been linked to pulmonary edema and is responsible for facilitating endothelial cell permeability [50]. In the present study, irradiation had no effect on IL8 secretion, regardless of timepoint, which may relate to the fact that the models used are simplified versions of complex tissues lacking systemic integration and that the observation time after irradiation was limited.

The full extent of IL8 and its role in RP remains somewhat controversial. Zhang and coworkers (2025) reported that patients who had higher IL8 serum levels prior to radiotherapy had a higher occurrence and risk of developing RP [51]. In a different study by Wang and coworkers (2017), it was reported that lower IL8 levels prior to radiation treatment were associated with increased risk of developing lung toxicity [52]. MCP1, also known as CCL2, is a chemokine that mediates recruitment and activation of immune cells [50]. MCP1 is secreted hours after radiation, with some studies reporting elevated levels even up to 26 weeks post radiotherapy [53]. In our lung models, MCP1 levels were not influenced by irradiation modality nor by the timepoint pIR.

IL6 is another pro-inflammatory cytokine responsible for stimulating growth and differentiation of lymphocytes. IL6 is secreted by alveolar macrophages, AT2 cells, but can also be produced in response to TNFα signaling [50]. Studies have shown that higher pre-treatment IL6 serum levels may predispose patients to radiation pneumonitis [54,55]. In this study, albeit statistically insignificant, we observed a clear upregulation trend with MRT-valley irradiation on D3, D6 and D21, while BB and MRT-EUD irradiation significantly increased IL6 levels on D21. The IL6 fold change in MRT-valley samples was similar to BB and MRT-EUD, which suggests a potentially similar inflammatory response, which could be confirmed with a larger sample size.

DSBs can lead to a formation of cytosolic DNA fragments, which act as damage-associated molecular patterns (DAMPs) and as such can be recognized by cGAS and further propagate the cGAS/STING cascade [56]. One of the downstream targets of cGAS/STING is nuclear factor kappa-light-chain-enhancer of activated B cells (NF-κB) [57]. Activation of NF-κB in macrophages leads to their polarization towards M1-like phenotype [58]. M1 macrophages secrete cytokines such as IL6, and are considered to be pro-inflammatory [59,60]. Considering that a significant IL6 upregulation was observed on D21, this suggests that there is an ongoing inflammation, which may not be favorable for the normal tissue. As mentioned above, unrepaired and persistent damage can be a trigger for cellular senescence and release of SASP secretory molecules, with one of them being IL6 [39,61]. Thus, a more thorough investigation would help to better understand to what extent AT2 cells or macrophages contribute to IL6 secretion, as well as to elucidate the role of cytokine in normal lung tissue. In response to pro-inflammatory stimuli (quartz particles), on day 21, a late upregulation of IL6 levels was documented by Barosova and coworkers (2020). This observation was made only when 3D EpiAlveolar™ models were co-cultured with monocyte-derived macrophages (MDM), whereas without MDMs an increase in released IL6 was observed at earlier timepoints, specifically on days 4 and 7 [18]. A follow-up study should investigate whether an earlier inflammatory response would also be observed with the experimental setup presented in this study in the absence of macrophages. Since macrophages play an important role in radiation-induced lung injury, future studies should also address their recruitment, activation status and polarization following broad-beam and MRT treatment.

In the context of lung cancer, treatment with MRT is currently largely in the preclinical stage. In vivo studies have demonstrated enhanced normal-tissue sparing and improved survival compared to BB irradiation [11,45]. However, recent analyses emphasize substantial heterogeneity in experimental design, dosimetry, and biological endpoints, underscoring the need for improved standardization [62]. Current investigations involving MRT irradiation of lung tumors are largely restricted to third-generation synchrotron facilities where ultra-high peak dose rates and minimal beam divergence can be achieved. At present, a compact X-ray tube is under development that would enable the translation and practical implementation of MRT into clinical settings [63].

In summary, our findings show that MRT-EUD is comparable with BB in context of DSB repair outcome, further supporting the perspective of MRT being able to achieve greater tumor control than conventional radiotherapy, while maintaining the same or lower normal-tissue damage [64]. Upregulation in IL6, a pro-inflammatory cytokine, was only observed 21 days after irradiation. Apart from a single BB-irradiated model showing fibrotic changes and extensive collagen deposition, none of the other irradiated lung tissue models exhibited a fibrotic response regardless of timepoint. The mechanisms behind MRT normal-tissue sparing are not fully understood; however, the fraction of tissue receiving valley and peak could potentially play an important role. Moreover, the valley regions are predominantly responsible for predicting normal-tissue toxicity [65]. Here, we utilized the 3D EpiAlveolar™ lung tissues for modeling the radiation response to both BB and MRT. Still, certain limitations exist. The tissue models were treated only with a single dose, which does not fully reflect the current radiotherapy treatment regimen for patients with lung cancer [26]. Hence, further investigation should include a more detailed dose–response analysis at more timepoints in order to compare effects in tissue models with therapeutic potential of MRT. Given that AT2 cells constitute a primary stem cell population in the lung, AT2 cell turnover following radiation should also be studied. Lastly, as observed in both BB and MRT-EUD irradiated models, the induced DSBs were resolved by D21. Thus, earlier timepoints should also be assessed to evaluate the DSB repair dynamics after MRT and BB in more detail, as well as to investigate the differences between peak and valley regions. Lastly, in our study, culturing the tissues for 21 days proved not to be optimal, as it led to a decrease in cell numbers and layers, and as such highlights the importance of using the 3D EpiAlveolar™ lung tissues for shorter periods of time.

## 5. Conclusions

The present study investigated the response of human 3D EpiAlveolar™ lung tissue models to BB and two MRT irradiation approaches (EUD and valley). Overall, we demonstrated that at D21, MRT-EUD achieved DSB repair outcomes comparable to BB. Despite persistent, unrepaired damage still being present on D21 in MRT-valley-irradiated tissues, we found no evidence of upregulation in EMT markers or collagen deposition. We observed the same tissue response for BB and MRT-EUD irradiation, suggesting that the doses used in this study were below the threshold necessary to induce fibrotic changes, or that the 21-day culture time failed to allow for inducing a full fibrotic response. It is worth mentioning that only one BB-irradiated model had extensive fibrotic tissue remodeling in the form of a collagen nodule. This highlights the potential of EpiAlveolar™ models for further investigation of radiation-induced fibrosis, although additional tests with a larger number of models and optimizations are necessary to fully elucidate whether the observed fibrosis in one model was induced by radiation. Since pulmonary fibrosis is a late effect and occurs only in up to 28% of lung cancer patients receiving RT [66], more prolonged culturing of the tissue models may be required to get further insights but seems out of reach due to cell loss over time. Further investigation should also address the cellular source and functional role of upregulated cytokine IL6, which we observed at the later timepoint, as a response of normal-tissue reaction to ionizing radiation.

## Figures and Tables

**Table 1:** Primary and secondary antibodies used for IF staining.

Antibody	Dilution	Company
**Primary antibodies**		
Anti-phospho-Histone H2A.X (Ser139) monoclonal antibody, clone JBW301	1:500	Merck (Darmstadt, Germany)
Anti-53BP1 antibody [EPR2172(2)]	1:500	abcam (Cambridge, UK)
Anti-alpha smooth muscle actin antibody [EPR5368]–Goat IgG (Chimeric)	1:300	abcam
Anti-vimentin antibody [RV202]	1:250	abcam
COL1A1 (E8F4L) XP^®^ Rabbit mAb	1:250	Cell Signaling Technology (Danvers, MA, USA)
**Secondary antibodies**		
Goat-anti-mouse-Cy5	1:300	Dianova (Hamburg, Germany)
Donkey-anti-rabbit-Fab-Cy3	1:1000	Dianova
Goat anti-mouse Alexa 488	1:500	Dianova

**Table 2:** Percentage (%) of cells displaying DSB damage, i.e., one or more 53BP1, γH2AX or colocalized foci on D3 and D21. Letters denote differences compared to the sham: ^a^ *p* < 0.01, ^b^ *p* < 0.0001.

	53BP1 D3	53BP1 D21	γH2AX D3	γH2AX D21	Colocalized D3	Colocalized D21
Sham	13.23	15.7	3.98	3.24	1.83	0.65
BB	26.5 ^a^	11.89	6.47	4.71	4.58 ^a^	1.21
MRT-EUD	37.76 ^b^	17.8	16.23 ^b^	5.33	12.01 ^b^	2.36 ^a^
MRT-valley	38.63 ^b^	35.03 ^b^	18.51 ^b^	8.25 ^a^	11.99 ^b^	3.80 ^b^

**Table 3:** Percentage of cells lost as a result of prolonged cell culture and radiation.

	D3 IR vs sham D3 (sh.D3)			D21 vs sham D3 (sh.D3)			
	BB D3/sh.D3	MRT-EUD D3/sh.D3	MRT-valley D3/sh.D3	shamD21/shD3	BB D21/sh.D3	MRT-EUD D21/sh.D3	MRT-valley D21/sh.D3
**Av. cells/mm relative to sham (%)**	85.96	86.29	77.29	72.23	49.41	52.07	52.15
**Cell loss on D3 relative to sham D3 (%)**	14.04	13.71	22.71	27.77	50.59	47.93	47.85
**Culture-induced cell loss on D21 (%)**				27.77	27.77	27.77	27.77
**IR-induced cell loss on D21 (%)**					22.82	20.16	20.08

## Data Availability

The original contributions presented in this study are included in the article/Supplementary Materials. Further inquiries should be directed to the corresponding authors.

## Supplementary Materials

The following supporting information can be downloaded at https://www.mdpi.com/article/10.3390/cells15060500/s1, Figure S1: 53BP1 and γH2AX foci distribution on D3. Figure S2: 53BP1 and γH2AX foci distribution on D21. Figure S3: Quantified TGF-β relative to sham at the same timepoint. Figure S4: Enhanced image details of Figure 5D.

## Abbreviations

The following abbreviations are used in this manuscript:

ANOVA	One-way analysis of variance
AT1	Alveolar epithelial cells type 1
AT2	Alveolar epithelial cells type 2
αSMA	Alpha smooth muscle actin
BB	Broad beam
CAF	Cancer-associated fibroblasts
CTC	Center-to-center
COL1A1	Collagen type 1 alpha 1
DAMPs	Damage-associated molecular patterns
DDR	DNA damage response
DSB	Double-strand break
ECM	Extracellular matrix
EMT	Epithelial–mesenchymal transition
EUD	Equivalent uniform dose
fc	Fold change
FPC	Foci per cell
Gy	Gray
IF	Immunofluorescence
IL6	Interleukin 6
IL8	Interleukin 8
IR	Irradiation
LQ	Linear quadratic
MCP1	Monocyte chemoattractant protein 1
MDM	Monocyte-derived macrophages
MRT	Microbeam radiation therapy
NFκB	Nuclear factor kappa-light-chain-enhancer of activated B cells
pIR	Post irradiation
PVDR	Peak-to-valley dose ratio
RF	Radiation fibrosis
RIF	Radiation-induced foci
RP	Radiation pneumonitis
RT	Radiotherapy
SABR	Stereotactic ablative radiotherapy
SASP	Senescence-associated secretory phenotype
TGF-β	Transforming growth factor beta
TNFα	Tumor necrosis factor alpha
2D	Two-dimensional
3D	Three-dimensional
4DCT	Four-dimensional computed tomography
