# Supplementary material for: Differential Cytokine and DNA Damage Response of Human Lung Tissue Models to Broad-Beam and Microbeam Radiotherapy
Source: Cells. 2026 Mar 11;15(6):500. doi: 10.3390/cells15060500 (PMC13025839; doi:10.3390/cells15060500)
Supplement: Supplementary file 1 [file cells-15-00500-s001.zip › cells-4171278-supplementary.pdf]

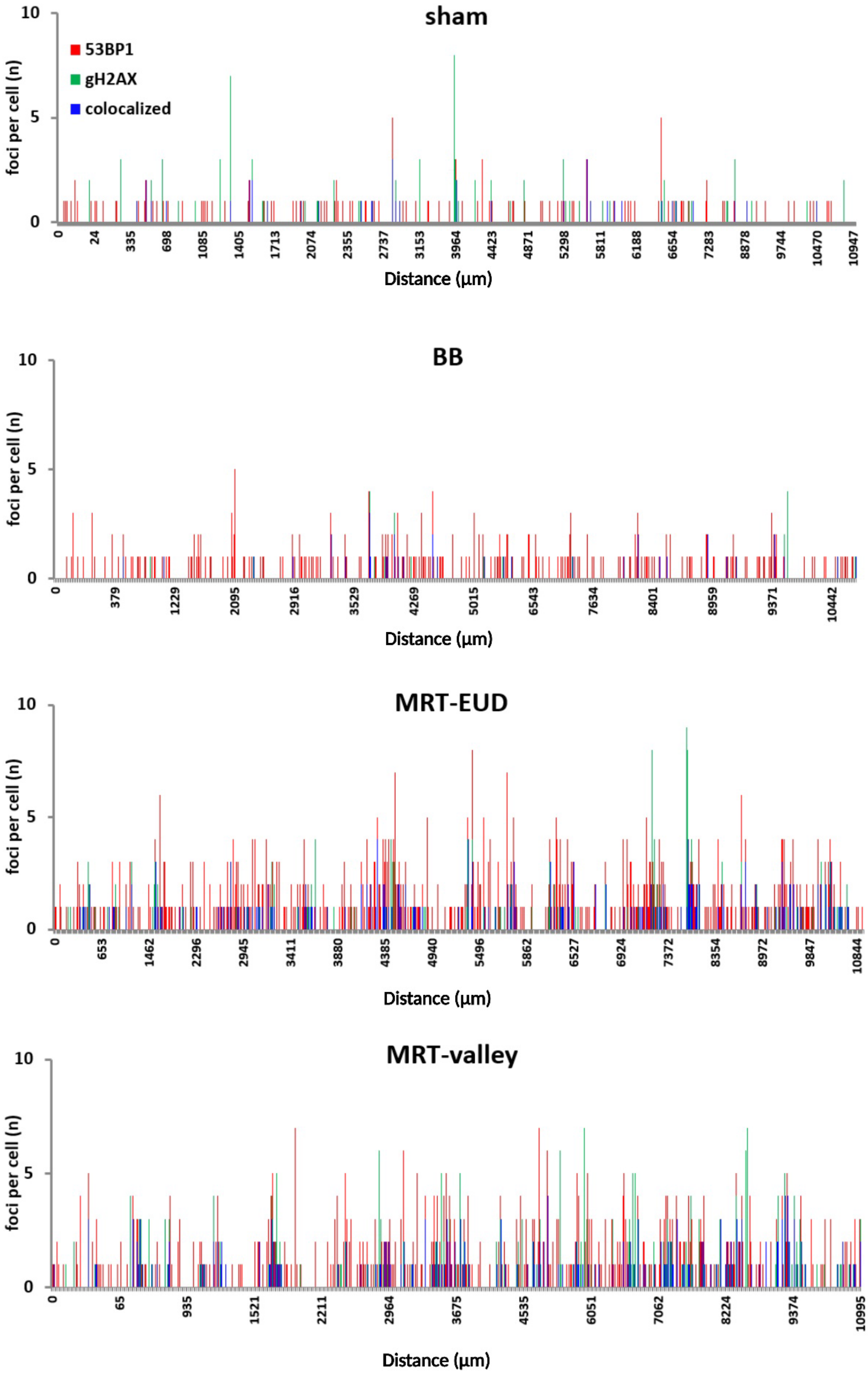

Supplementary Figure S1. 53BP1 and γH2AX foci distribution on D3.

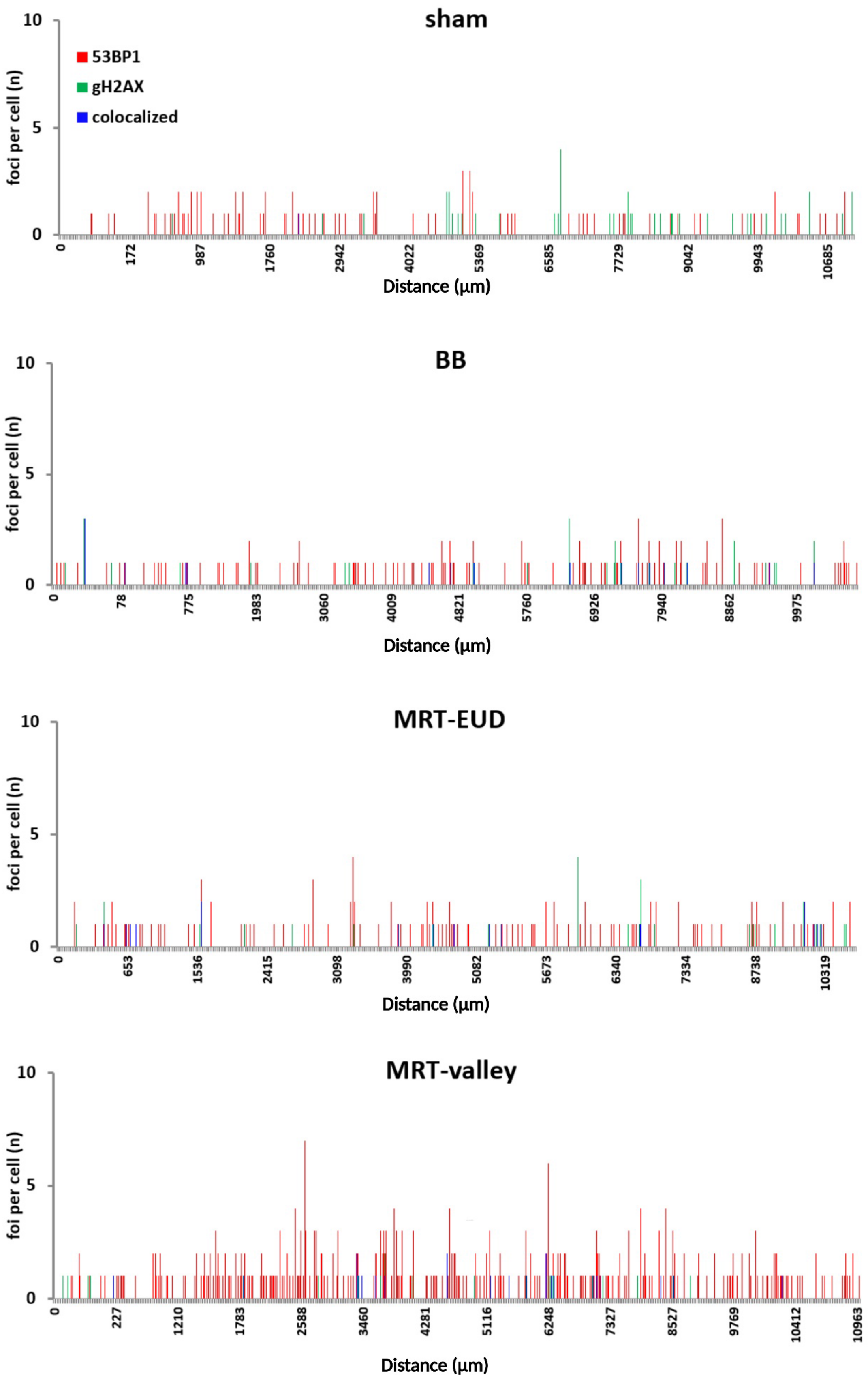

Supplementary Figure S2. 53BP1 and γH2AX foci distribution on D21

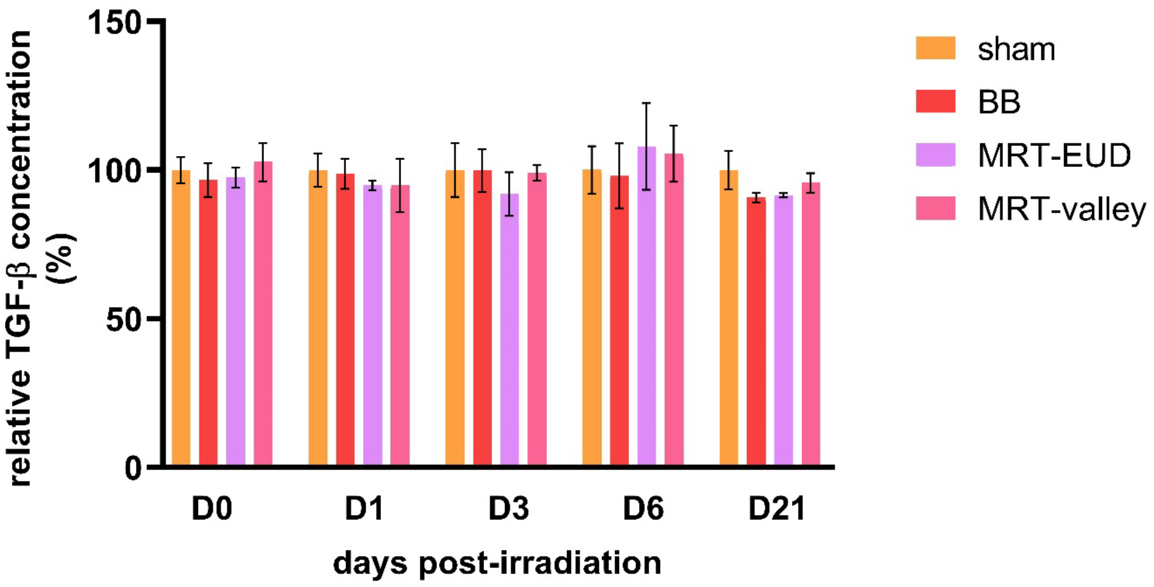

Supplementary Figure S3. Quantified TGF-β relative to sham at the same timepoint. Irradiated 3D lung tissue secretes baseline levels of TGF-β. Independently of timepoint and irradiation modality applied, secreted TGF-β levels in the culture medium were not significantly altered. Data are presented as mean ± SD of three biological replicates.

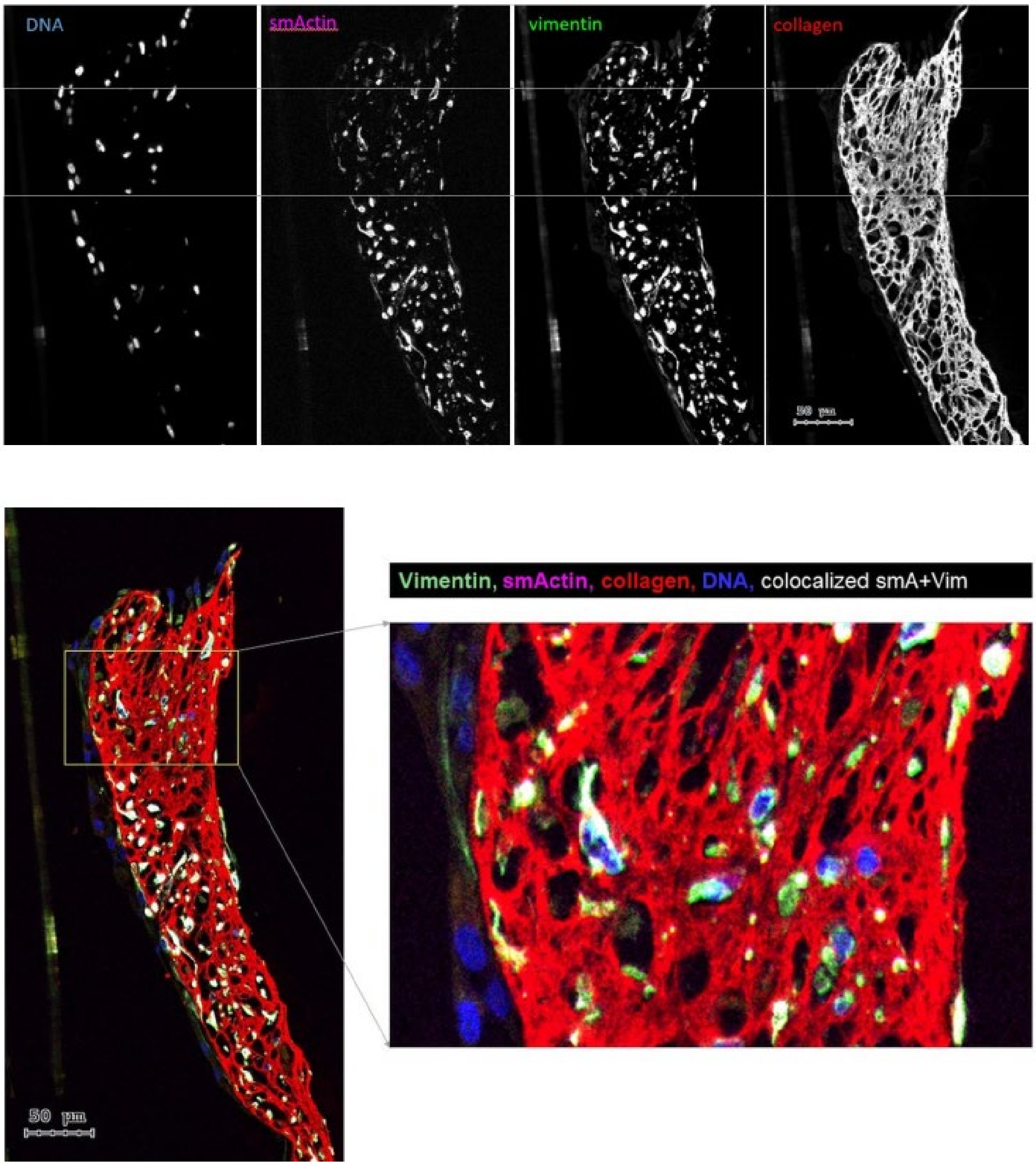

Supplementary Figure S4. Enhanced image details of Figure 5D. Upper row: single gray scale images of the different image channels for DNA (DAPI), smActin (Cy5), Vimentin (Alexa-488) and collagen (Cy3). Lower row: merge of the four channels and an enhanced detail (boxed in the left detail and above) showing whiteish colocalization of the cytoplasmic markers vimentin and smActin. Scale bars: 50 µm.
